# Review and Analysis of National Monitoring Systems for Antimicrobial Resistance in Animal Bacterial Pathogens in Europe: A Basis for the Development of the European Antimicrobial Resistance Surveillance Network in Veterinary Medicine (EARS-Vet)

**DOI:** 10.3389/fmicb.2022.838490

**Published:** 2022-04-07

**Authors:** Rodolphe Mader, Cristina Muñoz Madero, Birgit Aasmäe, Clémence Bourély, Els M. Broens, Luca Busani, Bénédicte Callens, Lucie Collineau, Paloma Crespo-Robledo, Peter Damborg, Maria-Eleni Filippitzi, William Fitzgerald, Annet Heuvelink, Jobke van Hout, Heike Kaspar, Madelaine Norström, Karl Pedersen, Tarja Pohjanvirta, Lucie Pokludova, Fabiana Dal Pozzo, Rosemarie Slowey, Cristiana Teixeira Justo, Anne Margrete Urdahl, Alkiviadis Vatopoulos, Christos Zafeiridis, Jean-Yves Madec, Jean-Philippe Amat

**Affiliations:** ^1^University of Lyon, French Agency for Food, Environmental and Occupational Health and Safety (ANSES), Laboratory of Lyon, Antibiotic Resistance and Bacterial Virulence Unit, Lyon, France; ^2^Agencia Española de Medicamentos y Productos Sanitarios (AEMPS), Coordinación del Plan Nacional Frente a la Resistencia a los Antibióticos (PRAN), Madrid, Spain; ^3^Institute of Veterinary Medicine and Animal Sciences, Estonian University of Life Sciences, Tartu, Estonia; ^4^Direction Générale de l’Alimentation, Bureau de la Santé Animale, Paris, France; ^5^Department of Biomolecular Health Sciences, Faculty of Veterinary Medicine, Utrecht University, Utrecht, Netherlands; ^6^Center for Gender-Specific Medicine, Istituto Superiore di Sanità, Rome, Italy; ^7^Antimicrobial Consumption and Resistance in Animals – AMCRA, Brussels, Belgium; ^8^University of Lyon, French Agency for Food, Environmental and Occupational Health and Safety (ANSES), Laboratory of Lyon, Epidemiology and Support to Surveillance Unit, Lyon, France; ^9^Department of Veterinary and Animal Sciences, University of Copenhagen, Frederiksberg, Denmark; ^10^Sciensano, Veterinary Epidemiology Unit, Belgian Research Centre for Health, Brussels, Belgium; ^11^Laboratory of Animal Production Economics, Department of Animal Production, Ichthyology, Ecology and Protection of the Environment, School of Veterinary Medicine, Aristotle University of Thessaloniki, Thessaloniki, Greece; ^12^Limerick Regional Veterinary Laboratory, Department of Agriculture, Food and the Marine, Limerick, Ireland; ^13^Royal GD, Deventer, Netherlands; ^14^Federal Office of Consumer Protection and Food Safety, Berlin, Germany; ^15^Norwegian Veterinary Institute (NVI), Ås, Norway; ^16^Department of Animal Health and Antimicrobial Strategies, National Veterinary Institute, Uppsala, Sweden; ^17^Finnish Food Authority, Veterinary Bacteriology and Pathology Unit, Helsinki, Finland; ^18^Institute for State Control of Veterinary Biologicals and Medicines (ISCVBM), Brno, Czechia; ^19^Department of Agriculture, Food and the Marine Laboratories, Celbridge, Ireland; ^20^Department of Public Health Policy, School of Public Health, University of West Attica, Athens, Greece; ^21^Ministry of Rural Development and Food, Athens, Greece

**Keywords:** antimicrobial resistance (AMR), monitoring, surveillance, Europe, animal, pathogen, veterinary, antibiotic

## Abstract

The monitoring of antimicrobial resistance (AMR) in bacterial pathogens of animals is not currently coordinated at European level. To fill this gap, experts of the European Union Joint Action on Antimicrobial Resistance and Healthcare Associated Infections (EU-JAMRAI) recommended building the European Antimicrobial Resistance Surveillance network in Veterinary medicine (EARS-Vet). In this study, we (i) identified national monitoring systems for AMR in bacterial pathogens of animals (both companion and food-producing) among 27 countries affiliated to EU-JAMRAI, (ii) described their structures and operations, and (iii) analyzed their respective strengths, weaknesses, opportunities and threats (SWOT). Twelve countries reported having at least one national monitoring system in place, representing an opportunity to launch EARS-Vet, but highlighting important gaps in AMR data generation in Europe. In total, 15 national monitoring systems from 11 countries were described and analyzed. They displayed diverse structures and operations, but most of them shared common weaknesses (e.g., data management and representativeness) and common threats (e.g., economic vulnerability and data access), which could be addressed collectively under EARS-Vet. This work generated useful information to countries planning to build or improve their system, by learning from others’ experience. It also enabled to advance on a pragmatic harmonization strategy: EARS-Vet shall follow the European Committee on Antimicrobial Susceptibility Testing (EUCAST) standards, collect quantitative data and interpret AMR data using epidemiological cut-off values.

## Introduction

The monitoring of antimicrobial resistance (AMR) in bacterial pathogens of animals (i.e., in clinical isolates from diseased animals) is not currently coordinated at European level. To fill this gap, experts of the European Union Joint Action on AMR and Healthcare Associated Infections (EU-JAMRAI),^1^ which aims to strengthen the European One Health strategy to tackle AMR (European Commission, 2017), recommended building the European Antimicrobial Resistance Surveillance network in Veterinary medicine (EARS-Vet) (Mader et al., 2021a). The objectives of EARS-Vet shall be to report on the current AMR situation, follow AMR trends and detect emerging AMR in bacterial pathogens of animals in Europe. The information generated would contribute to: (i) advise policy makers on interventions to mitigate AMR, taking the One Health approach, (ii) monitor the impact of European efforts to tackle AMR in the animal sector, (iii) support antimicrobial stewardship initiatives, especially the development of antimicrobial treatment guidelines in veterinary medicine, (iv) evaluate or revise marketing authorizations of antimicrobials, (v) generate epidemiological cut-off values (ECOFFs) and clinical breakpoints for the interpretation of antimicrobial susceptibility testing (AST) results, (vi) assess the risk of AMR transmission between animals and humans via non-food related routes, e.g., by direct contact between humans and companion or food-producing animals, and (vii) estimate the burden of AMR in animal health, e.g., attributable deaths and morbidity caused by infections with antimicrobial-resistant bacteria in animals.

In the One Health approach, EARS-Vet should be designed to complement and integrate with existing European monitoring systems for AMR, i.e., the European Antimicrobial Resistance Surveillance Network (EARS-Net) (European Centre for Disease Prevention and Control, 2020) and the European Food- and Waterborne Diseases and Zoonoses Network (FWD-Net) in the human sector (European Centre for Disease Prevention and Control, 2016), as well as the AMR monitoring in zoonotic and indicator bacteria, coordinated by the European Food Safety Authority (EFSA), which covers healthy food-producing animals (European Food Safety Authority and European Centre for Disease Prevention and Control, 2021).

It was also agreed by EU-JAMRAI experts that EARS-Vet should work as a European network of national monitoring systems (Mader et al., 2021a). Thus, in a bottom-up approach, an important step consisted of reviewing and analyzing existing national monitoring systems in Europe to allow for the development of an EARS-Vet framework that considers what is relevant and feasible to monitor in countries, and to advance on a harmonization strategy. A definition of the EARS-Vet scope, i.e., the combinations of animal species, production types, bacterial species, clinical specimens, and antimicrobials to be monitored in EARS-Vet was made by Mader et al. (2022). In brief, it covers cattle, swine, chicken, turkey, cats, and dogs; major bacterial pathogens of these animal species (*Escherichia coli, Klebsiella pneumoniae, Mannheimia haemolytica, Pasteurella multocida, Actinobacillus pleuropneumoniae, Staphylococcus aureus, Staphylococcus pseudintermedius, Staphylococcus hyicus, Streptococcus uberis, Streptococcus dysgalactiae*, and *Streptococcus suis*); and relevant antimicrobials for their treatment (e.g., tetracyclines, aminopenicillins, sulfonamide/trimethoprim), complemented with antimicrobials of more specific public health interest (e.g., carbapenems, tigecycline). Although reviews of AMR monitoring systems in the animal sector have already been published (Schrijver et al., 2017; Mesa Varona et al., 2020), they did not focus on clinical animal isolates, did not consider companion animals and provided limited information on the structures and operations of systems.

To continue the development of the EARS-Vet framework, and support the establishment, improvement and harmonization of national monitoring systems for AMR in animal bacterial pathogens, the present study aimed to (i) identify existing national monitoring systems for AMR in bacterial pathogens of animals among 27 countries affiliated to EU-JAMRAI (as potential future EARS-Vet participating countries), (ii) describe their structures and operations, and (iii) analyze their respective strengths, weaknesses, opportunities and threats (SWOT).

## Materials and Methods

### Identification of National Monitoring Systems for Antimicrobial Resistance in Bacterial Pathogens of Animals

EU-JAMRAI stakeholders from 27 countries of the EU/European Economic Area (EEA) were contacted in 2018–2020 and asked if a national monitoring system for AMR in bacterial pathogens of animals was in place in their country: Austria, Belgium, Croatia, Cyprus, the Czech Republic, Denmark, Estonia, Finland, France, Germany, Greece, Hungary, Italy, Ireland, Latvia, Lithuania, Malta, the Netherlands, Norway, Poland, Portugal, Romania, Slovakia, Slovenia, Spain, Sweden, and the United Kingdom. A national monitoring system for AMR in bacterial pathogens of animals was defined as any system collecting and regularly analyzing AST results produced on bacterial isolates from clinical samples of animals that can be considered as having a national coverage. However, no criteria were established on geographic data representativeness or scope of bacterial and animal species.

### Description and Performance Analysis of National Monitoring Systems for Antimicrobial Resistance in Bacterial Pathogens of Animals

Stakeholders in individual countries who reported having a national monitoring system for AMR in bacterial pathogens of animals were invited to have their system described and performance analyzed, using a common methodology across participating countries.

The description of existing national AMR monitoring systems was done through a questionnaire covering the following key areas: (i) political and financial support, (ii) monitoring objectives, (iii) central institutional organization, (iv) laboratory network, (v) monitoring procedures, (vi) laboratory techniques, (vii) monitoring data, (viii) communication and (ix) evaluation (Supplementary Table S1).

The performance analysis of national monitoring systems was done using SWOT (strengths, weaknesses, threats, and opportunities) analyses (Renault, 2021). In countries where several national systems were identified, only one SWOT analysis was carried out, to assess the overall picture at national level.

Both questionnaires and SWOT analyses were completed in 2019–2020 during 1-day physical meetings, except for one country (Estonia), where two virtual meetings were organized due to travel restrictions linked to the COVID-19 pandemic. Each physical or virtual meeting was jointly organized by the French Agency for Food, Environmental and Occupational Health and Safety (ANSES) and the EU-JAMRAI partner institution(s) of the visited country. Participants were relevant national stakeholders, including the coordinator(s) and key experts of the national AMR monitoring system, as well as an expert from ANSES, who asked the questionnaire and facilitated the completion of the SWOT analyses. Frequently reported topics in SWOT analyses were identified *a posteriori* and used to describe the main strengths, weaknesses, threats and opportunities of national monitoring systems. After completing the questionnaires and SWOT analyses, further clarifications were obtained via email exchanges on an *ad hoc* basis between the expert from ANSES and the coordinators of the national monitoring systems. Questions considered of lower relevance or not leading to factual answers were excluded from the data analysis.

## Results

### Identification of National Monitoring Systems for Antimicrobial Resistance in Bacterial Pathogens of Animals

**FIGURE 1:**
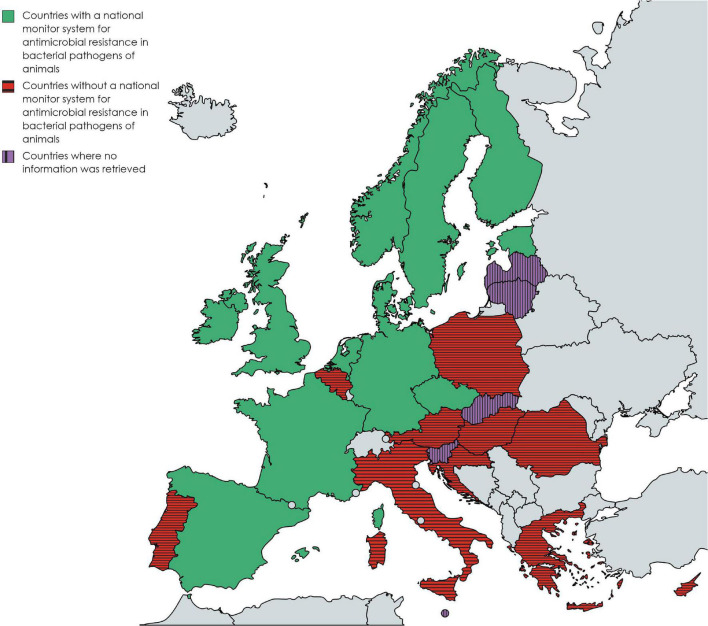
Map of Europe showing the countries that have at least one national monitoring system for antimicrobial resistance in bacterial pathogens of animals, do not have no such system, or where information was missing, among countries affiliated to the European Union Joint Action on Antimicrobial Resistance and Healthcare Associated Infections (EU-JAMRAI) and as of 2020. Created with mapchart.net.

The following 12 countries reported having a national monitoring system for AMR in bacterial pathogens of animals: the Czech Republic, Denmark, Estonia, Finland, France, Ireland, Germany, the Netherlands, Norway, Spain, Sweden, and the United Kingdom. Stakeholders from Denmark, the Netherlands and Sweden reported more than one monitoring system in their countries (Table 1). Ten countries reported not to have such a system (Austria, Belgium, Croatia, Cyprus, Greece, Hungary, Italy, Poland, Portugal, and Romania). However, contacted experts in Italy reported that AMR monitoring in diseased animals was organized in some regions (e.g., Friuli Venezia Giulia). In Belgium, two veterinary diagnostic laboratories performed independent regional AMR monitoring (ARSIA, 2017; Dierengezondheidszorg Vlaanderen, 2019). No information was retrieved from Latvia, Lithuania, Malta, Slovakia, and Slovenia. Figure 1 shows on a map of Europe which countries have a monitoring system (at least one), no system, or where information is missing among countries affiliated to the EU-JAMRAI.

**TABLE 1:** List of 15 national monitoring systems for antimicrobial resistance in bacterial pathogens of animals described and analyzed in the study.

Country	Name of the national monitoring system
Finland	Finnish Veterinary Antimicrobial Resistance Monitoring and Consumption of Antimicrobial Agents (FINRES-Vet)
Sweden	Swedish Veterinary Antibiotic Resistance Monitoring (Svarm)
Sweden	Swedish Veterinary Antibiotic Resistance Monitoring—farm animal pathogens (SvarmPat)
The Czech Republic	Czech National Monitoring of Target Pathogens’ Antimicrobial Resistance (CZ NMTP)
Norway	Norwegian Monitoring Program for Antimicrobial Resistance in bacteria from feed, food and animals (NORM-VET)
Denmark	Technical University of Denmark / Danish Veterinary and Food Administration (DTU/VFA)*^/^**
Denmark	University of Copenhagen (UC)*
Denmark	Agricultural knowledge and innovation center (SEGES)*
The Netherlands	University of Utrecht (UU)*
The Netherlands	GD Animal Health Surveillance System
Germany	National Resistance Monitoring in Bacterial Pathogens of Animals (GE*RM*-Vet)
Ireland	Department of Agriculture, Food and the Marine (DAFM)*
Spain	Spanish Antimicrobial Resistance Surveillance in Clinical Animal Pathogens (*Sistema Español de Vigilancia de Animales Enfermos*—SEVAE)
Estonia	Veterinary and Food Laboratory / University of Life Sciences (VFL/ULS)*
France	French surveillance network for antimicrobial resistance in diseased animals (RESAPATH)

*Names of coordinating institutions were used to identify monitoring systems without official name for the purpose of this study. **During 2020, under the administration of the VFA, the coordination of this monitoring system has been gradually taken over by the Statens Serum Institut and the University of Copenhagen. DTU does however still supply data, e.g., for cattle pathogens.

The questionnaire and SWOT analysis were completed for all countries identified with a national monitoring system for AMR in diseased animals, except the United Kingdom, where the country visit got canceled due to the COVID-19 pandemic and could not be rescheduled virtually within the project time frame. In total, 15 monitoring systems from 11 countries were described and analyzed (Table 1).

### Description of National Monitoring Systems for Antimicrobial Resistance in Bacterial Pathogens of Animals

Results are presented per each key area, as defined in section “Materials and Methods.”

#### Political and Financial Support

All countries under investigation had a National Action Plan (NAP) to tackle AMR. All NAPs, except in the Netherlands and Estonia, specify the need to perform AMR monitoring in diseased animals and often request specific organizations to perform this monitoring. However, as shown in Supplementary Table S2, only two countries have set up a regulated system, i.e., AMR monitoring is enforced by law: Germany, via its Medicinal Products Act (Bundesministerium der Justiz und für Verbraucherschutz, 1976) and the Czech Republic, via its Resolution no. 75 on the Action Plan of the National Antibiotic Programme of the Czech Republic for period 2019–2022 (Government of the Czech Republic, 2019).

Even if not regulated or not integrated in NAPs, national monitoring systems can still benefit from governmental support such as in the Netherlands, where AMR monitoring is funded by the Dutch government (for companion animals) or by both the government and producer/interbranch organizations (for livestock). Still, some national monitoring systems remain independent initiatives such as the monitoring of the University of Copenhagen (UC) in companion animals, without dedicated governmental or specific financial support (AST cost is supported by animal owners) (Supplementary Table S2).

When dedicated national funding is allocated, it can consist of a subsidy (i) to decrease the AST costs for farmers (in the Czech Republic and Spain) or (ii) to do more pathological examinations on farm animals, which are, ultimately, an important source of AST data (GD Animal Health Surveillance System). In this way, subsidies support two goals: collecting more monitoring data and supporting veterinary antimicrobial stewardship. In some countries (Norway and Germany), a budget has been allocated to re-test isolates at a central laboratory. This ensures AST harmonization, irrespective of isolate origin, in terms of method, antimicrobials tested, and interpretive criteria applied. It also enables to test antimicrobials which are not tested by field veterinary diagnostic laboratories, but which may be of interest in a public health perspective (e.g., carbapenems). Alternatively, funding can also be intended to complementary monitoring programs to fill gaps in the regular monitoring in bacterial pathogens of animals [e.g., the Swedish Veterinary Antibiotic Resistance Monitoring (Svarm) is complemented by a farm animal pathogen monitoring program (SvarmPat)]. Other costs of AMR monitoring, such as human resources of the coordination team, may either be supported by the regular budget of the coordinating institution or by dedicated funding (e.g., hours for collecting, processing and reporting data in the GD Animal Health Surveillance System are subsidized via the Dutch animal Health monitoring) (Supplementary Table S2).

#### Monitoring Objectives

Monitoring objectives are summarized in Table 2 and available by national monitoring system in Supplementary Table S3.

**TABLE 2:** Objectives of the 15 reviewed national monitoring systems for antimicrobial resistance in bacterial pathogens of animals.

Monitoring objectives	Number of national monitoring systems (out of 15)
A. Monitoring AMR trends in animal bacterial pathogens to antimicrobials of interest for veterinary medicine	14
B. Detecting AMR emergences in animal bacterial pathogens to antimicrobials of interest for veterinary medicine	13
C. Monitoring AMR trends in animal bacterial pathogens to antimicrobials of interest for public health*	12
D. Detecting AMR emergences in animal bacterial pathogens to antimicrobials of interest for public health*	12
E. Informing veterinarians on AMR levels in animal bacterial pathogens, to help them in their antimicrobial treatment decisions	13
F. Developing antimicrobial therapy guidelines intended to veterinarians	6
G. Monitoring the impact of the National Action Plan (along with other possible indicators such as antimicrobial use data)	5
H. Advising policy makers on interventions to mitigate AMR	7
I. Better understanding the AMR epidemiological links between the animal and human sectors	6
J. Better understanding the AMR epidemiological links between the animal and environmental sectors	3
K. Better understanding the links between AMR and antimicrobial use data	8
L. Assessing the risk of AMR transmission between animals and humans via non-food related routes (e.g., by direct contact between humans and companion or food-producing animals)	7
M. Estimating the burden of AMR in animal health (e.g., attributable deaths and morbidity caused by infections with antimicrobial-resistant bacteria in animals)	0
N. Other monitoring objective	0

*Many antimicrobials are of interest to both animal and public health. This objective was ticked only if the monitoring system does aim to provide useful AMR data in a public health perspective.

Nearly all systems aim to monitor AMR trends (14/15) and detect emerging AMR (13/15) for antimicrobials of interest to veterinary medicine. Although 13 monitoring systems aim to inform veterinarians on AMR levels to help them in their antimicrobial treatment decisions, only six systems produce AMR data with the specific aim to develop antimicrobial therapy guidelines intended to veterinarians. It is worth noting that the Czech National Monitoring of Target Pathogens’ Antimicrobial Resistance (CZ NMTP) was established primarily to support antimicrobial stewardship by incentivizing the use of AST and evidence-based antimicrobial therapy by veterinarians.

Most systems (12/15) also aim to monitor AMR trends and detect emerging AMR in a public health perspective, demonstrating the frequent adoption of the One Health approach. Furthermore, in seven systems such AMR data are intended to be used to assess the risk of AMR transmission between animals and humans via non-food related routes, as well as (in six systems) to better understand AMR epidemiological links between the animal and human sectors.

Although most NAPs include AMR monitoring in diseased animals (see *Political and Financial Support*), only seven and five systems produce AMR data with the specific objectives to advise policy makers on interventions to mitigate AMR and to monitor the impact of the NAP, respectively.

#### Central Institutional Organization

A steering committee was defined as a committee defining the vision and objectives of a monitoring system, as well as approving monitoring procedures (proposed by the coordination team or an *ad hoc* scientific and technical committee). As shown in Supplementary Table S2, five national monitoring systems have such a steering committee in place: CZ NMTP, the Spanish Antimicrobial Resistance Surveillance in Clinical Animal Pathogens (*Sistema Español de Vigilancia de Animales Enfermos*—SEVAE), the French surveillance network for antimicrobial resistance in diseased animals (RESAPATH), the GD Animal Health Surveillance System and the national monitoring system coordinated by the Technical University of Denmark and the Danish Veterinary and Food Administration (DTU/VFA). They show various compositions, ranging from representatives of a single organization to representatives of diverse institutions from both public and private sectors (e.g., both private and public laboratories in RESAPATH).

Coordinating institutions, defined as those running national monitoring systems in practice (i.e., interacting with data providers, collecting data, analyzing data, writing reports etc.), are also very different across systems (Supplementary Table S2). They may be private [e.g., the agricultural knowledge and innovation center (SEGES) in Denmark or Royal GD in the Netherlands] or public (e.g., universities, food safety agencies, medicine agencies).

Sweden, Denmark, and the Netherlands have more than one monitoring system for AMR in clinical animal isolates, but these are only integrated in Sweden. However, collaboration between systems usually exists, such as in Denmark, where SEGES provides AST data from swine to DTU/VFA.

#### Laboratory Network

As shown in Supplementary Table S4, seven systems perform AMR monitoring on isolates collected from a single laboratory. Other systems are based on a network of laboratories, usually made up of a limited number of laboratories, except RESAPATH, the German National Resistance Monitoring in Bacterial Pathogens of Animals (GE*RM*-Vet) and SEVAE, which operate through 71, 30, and 22 laboratories, respectively. Networked laboratories either belong to the same institution [e.g., the Department of Agriculture, Food and the Marine (DAFM) in Ireland], or comprise independent laboratories, belonging to both private and public sectors (e.g., SEVAE and RESAPATH) or with a joint public/private status (CZ NMTP). The National Reference Laboratory (NRL) for AMR in the animal sector is involved in six systems.

All laboratory networks, except in SEVAE, include one or two central laboratories. In RESAPATH, the two central laboratories (both belonging to ANSES) are responsible for AMR monitoring in different animal species. Central laboratories may have diverse missions, depending on the system: analysis of diagnostic specimens, re-testing isolates of specific interest from field laboratories, re-testing and performing complementary molecular analyses on isolates with specific phenotypes and organizing proficiency testing (PT) on AST as part of reference activities (Supplementary Table S4).

#### Monitoring Procedures

All systems are primarily based on a passive monitoring procedure, i.e., AST results come from samples, that are routinely submitted by field veterinarians. However, two of them also include an active monitoring component (SvarmPat and DTU/VFA system), where diseased animals are sampled specifically for the purpose of AMR monitoring (and would likely not be sampled otherwise). These active monitoring components aim to fill knowledge gaps and generate more representative AMR data than by passive monitoring. They remain project-based and monitoring priorities can change over time.

#### Laboratory Techniques

Laboratory techniques and standards of the 15 monitoring systems are described in Supplementary Table S5.

Regarding bacterial identification, the most commonly used method was the Matrix Assisted Laser Desorption Ionization—Time of Flight (MALDI-TOF) (by 14/15 systems), followed by Analytical Profile Index (API) galleries (by 3/15 systems). Among national monitoring systems based on a laboratory network and which do not re-identify all bacterial species at a central laboratory, SEVAE, CZ NMTP, and the DAFM system have developed agreements or standard operating procedures, so that all member laboratories use the same bacterial identification method. In CZ NMTP and the DAFM system, PT is also in place to ensure the performance of participating laboratories in bacterial identification. Conversely, no standard or recommendation is provided to field laboratories in RESAPATH and the Finnish Veterinary Antimicrobial Resistance Monitoring and Consumption of Antimicrobial Agents (FINRES-Vet).

Regarding AST, broth microdilution is the reference method in 12 systems, followed by disk diffusion for four systems. Both methods are used as part of FINRES-Vet, depending on the animal species. Broth microdilution is also used in the DAFM system for confirmatory testing and in the Estonian system, coordinated by the Veterinary and Food Laboratory and the University of Life Sciences (VFL/ULS), for testing colistin resistance. Eight countries follow the standards of the Clinical and Laboratory Standards Institute (CLSI) and interpret results according to its veterinary clinical breakpoints (when available). Different alternatives are used by countries when veterinary breakpoints are not available (a situation reported as frequent): epidemiological cut-off values (ECOFFs) of the European Committee on Antimicrobial Susceptibility Testing (EUCAST), epidemiological cutoff values (ECVs) of CLSI, human clinical breakpoints of EUCAST or CLSI, ECOFFs of the veterinary guidelines of the Antibiogram Committee of the French Society of Microbiology (CA-SFM), clinical breakpoints suggested by pharmaceutical companies, minimum inhibitory concentration (MIC) 90%, or internal ECOFFs (i.e., determined on internal MIC distributions). On the other hand, NORM-Vet and Svarm/SvarmPat follow the EUCAST guidelines and its ECOFFs (when available), while RESAPATH follows the AST technique and ECOFFs (when available) recommended in the veterinary guidelines of CA-SFM. In case of missing ECOFFs from their respective standards, RESAPATH does not provide any interpretation, NORM-Vet and Svarm/SvarmPat use internal ECOFFs (when possible) or CLSI ECVs (when available), while Svarm/SvarmPat may also use CLSI clinical breakpoints.

For the six systems that operate on a laboratory network without systematically re-testing all isolates centrally (FINRES-Vet, CZ NMTP, DTU/VFA system, DAFM system, SEVAE, and RESAPATH), a single AST standard is used. To guarantee the quality of AST results (as well as harmonization when there is a laboratory network and no central re-testing of all isolates), laboratories are either accredited on the AST technique and/or participate in PT in 13 systems. PT can be organized by the NRL, the central laboratory, or an institution from a different country, such as the VETQAS^®^, proposed by the Animal and Plant Health Agency in the United Kingdom. Other quality control measures like the use of reference strains with a defined MIC range are also in place, hence all systems have some level of quality control.

#### Monitoring Data

Monitoring data are presented in Supplementary Table S6. Animal species, bacterial species, antimicrobial and AST result are pieces of information collected by all systems. Data on specimens are collected in 12/15 systems and production type in 7/13 monitoring systems (excluding the two systems focusing on companion animals). Six systems collect information on prior antimicrobial treatment or reason for performing AST. National monitoring systems collect different volumes of data, from a few hundreds to about 55,000 isolates per year. The coordinating institutions own their data, except in CZ NMTP and SEVAE, where data are the property of farmers. All national systems collect quantitative AST data (i.e. not only interpreted results).

#### Communication

As shown in Supplementary Table S7, for 12 systems, data are analyzed every year leading to the publication of an annual report. For those systems, there is a lag of 4 months to 1 year from the production of AST data to the reporting for the respective calendar years. In the SEGES system and GD Animal Health Surveillance System, data analysis is carried out more frequently (up to four times a year), with proportions of resistance calculated and reported on their website, so that more timely information can be used by antimicrobial prescribers and users. Targeted audience for these reports usually consists of veterinarians, veterinary diagnostic laboratories, relevant governmental bodies, farming organizations, AMR experts and organizations responsible for the development of treatment guidelines.

#### Evaluation

RESAPATH and the DAFM system were the only two monitoring systems which had been evaluated. RESAPATH was evaluated twice (in 2010 and 2018), using the *Outil d’Analyse des Systèmes d’Information en Santé* (OASIS) (Mader et al., 2021b). For the DAFM system, this was done once in 2019, as part of a country visit by ECDC and the Directorate-General for Health and Food Safety, to support the development and implementation of the Irish strategy for tackling AMR based on a “One Health” approach (European Centre for Disease Prevention and Control and Directorate-General for Health and Food Safety, 2019). Only RESAPATH monitors performance indicators every year (listed in Supplementary Table S8), such as the *Proportion of laboratories obtaining a score above or equal to 31/36 at the AST PT organized by ANSES*, which should be of at least 95%.

### Strengths, Weaknesses, Opportunities and Threats Analyses

Eleven SWOT analyses were produced (one per country), available in Supplementary Table S9. Nine themes were frequently reported and used to describe and analyze the results: (1) Public awareness and Policies, (2) Flexibility and Utility, (3) Data access and Sampling, (4) Data management and Analysis, (5) Harmonization, (6) Representativeness, (7) Geographical coverage, (8) Collaboration and Integrated monitoring, and (9) Sustainability and Resources.

#### Strengths

The **Flexibility and Utility** of the system were reported as a major strength in nine countries out of 11. All of them highlighted their system had a broad scope, making it possible to monitor a diverse range of animal species, bacterial species and antimicrobial combinations, of relevance to the local epidemiological situation. Utility for veterinary practice (through the production of antimicrobial treatment guidelines or other educational material targeting veterinary professionals and farmers) was reported as a strength by three countries. Another frequently reported strength (*n* = 10) was the level of **Harmonization** of the AST analyses performed within the monitoring system, with most participating laboratories being accredited for AST. **Geographical coverage** was reported as good (*n* = 5) or unknown (*n* = 1).

Most participating countries (*n* = 9) reported that a strength of their system was the very good collaboration they had with other AMR monitoring stakeholders or partners, including those in the human sector (*n* = 5) and in healthy animals (*n* = 3), ministries of agriculture (*n* = 3), veterinary research institutes (*n* = 2), or private industry (*n* = 1) **(Collaboration and Integrated monitoring)**. Five countries also reported their system relied on a small team, thereby facilitating internal communication and coordination.

#### Weaknesses

**Data management and Analysis** appeared as a major weakness across participating countries. Five countries reported a lack of efficient data management tools (e.g., for data cleaning and data extraction), while three countries reported poor data quality, such as incomplete or invalid metadata (e.g., prior antimicrobial use or age category). Capacity for storing isolates collected through the monitoring system was reported as an issue in only one country.

Another weakness shared across systems was the **Representativeness**, i.e., the ability to provide a reliable picture of AMR through sufficient and representative data for each combination of animal species/bacteria, which was reported as low (*n* = 9) or unknown (*n* = 2) in most countries. In most systems, this was related to the use of passive sample collections to monitor AMR.

#### Opportunities

Most participating countries (*n* = 8) considered the societal context, in terms of **Public awareness and Policies**, as an opportunity for their monitoring system. Six countries mentioned an increasing level of public awareness on AMR, and five reported AMR was a policy priority in their country. Three countries claimed their system was supported by national or EU Action plans on AMR, and four countries had national legislation or programs in place to enforce or encourage the use of AST in the veterinary sector, thus contributing to an increase in the volume of data submitted to the monitoring system.

While collaboration between sectors was perceived as a strength in most participating countries, four countries reported there was an opportunity to improve the One Health integration of their system **(Collaboration and Integrated monitoring)**. Six countries also reported EARS-Vet as an opportunity to learn about practices and solutions from other countries that could be applicable to their own system.

#### Threats

Almost all participating countries (*n* = 9) reported the economic vulnerability as the main threat to their monitoring system, with a regular decrease in available financial and human resources **(Sustainability and Resources)**. Three countries reported that lack of funding prevented them from performing more advanced data analyses or improving data representativeness. Of note, two countries also reported a lack of skilled AMR experts in animal health in their country.

Various threats to **Data access and Sampling** were also reported, including issues around samples sent abroad for AST (*n* = 3), or the use of alternative techniques (e.g., PCR or rapid AMR detection tests) preventing access to more conventional AST results (*n* = 2). The development of large and competitive private laboratory companies was perceived as a threat in five countries, although three countries had good collaborations with these laboratories, and perceived them as an opportunity to further expand their laboratory network **(Collaboration and Integrated monitoring)**.

## Discussion

To our knowledge, this is the first review of national monitoring systems for AMR in bacterial pathogens of animals describing system structures and operations and analyzing their performance with a standardized approach. We identified 12 countries with at least one national monitoring system, among 27. This led to a description and analysis of 15 systems in 11 European countries thanks to a questionnaire and SWOT analyses.

A strength of our approach was to include in the study almost all countries of the EEA (only Iceland, Bulgaria, Liechtenstein and Luxemburg are missing). This was facilitated by the EU-JAMRAI network of partner institutions and collaborating stakeholders, which facilitated contact with AMR experts in 27 countries. It enabled the identification of monitoring systems, which may not be easily identified through online searches, when no report is publicly available or only available in local language (as in Estonia, the Czech Republic and in Spain). However, no information could be retrieved for Latvia, Lithuania, Malta, Slovakia and Slovenia, despite several attempts to establish collaboration through the network. In addition, the questionnaire and SWOT analysis were not completed for the United Kingdom.

The survey questionnaire allowed the provision of a broad overview of the structure and operations of national monitoring systems, by covering areas, which were not limited to microbiology, but also funding, regulation, institutional organization, procedures, laboratory networks, communication, and evaluation. Various methods exist to assess the performance of monitoring systems (Calba et al., 2015), but a simple one was adopted in this study, i.e., SWOT analyses. This was a pragmatic choice, to be able to collect key information in a short amount of time, considering that 15 monitoring systems from 11 countries were included. Still, as SWOT analyses were completed by relevant national stakeholders, including the coordinator(s) of the evaluated monitoring system, collected information could be biased. However, this risk was considered low because the aim was not to give a score and compare the performance of monitoring systems, and because of the presence of an external facilitator who previously administered the questionnaire to describe the system.

### Identification of National Monitoring Systems for Antimicrobial Resistance in Bacterial Pathogens of Animals

The review clarified gray areas reported in a previous literature review (Schrijver et al., 2017), by confirming the absence of AMR monitoring system in diseased animals in Romania and Croatia, and by confirming that the VAV system in Spain and ITAVARM in Italy are no more in operation. Moreover, previous reviews indicated inconsistent results regarding the existence of an AMR monitoring system covering clinical animal isolates in Poland (Ferreira and Stärk, 2017; Schrijver et al., 2017). In this study, contacted experts in Poland indicated there was no such monitoring.

On the one hand, many Southern and Eastern European countries do not have any national monitoring system for AMR in bacterial pathogens of animals (Figure 1), emphasizing a major gap in AMR monitoring in Europe. The reasons why so many countries do not have a monitoring system in place were not specifically explored. However, as part EU-JAMRAI, SWOT analyses were also carried out in Belgium, Greece and Italy, to help define a strategy to establish a national monitoring system. The weaknesses and threats identified during this exercise suggest that the reasons could include the (i) lack of dedicated resources, (ii) lack of harmonization between laboratories, (iii) difficulties to federate field diagnostic laboratories in the absence of a legal framework, (iv) difficulties to collect AMR data passively when veterinarians rarely request AST and (v) difficulties to reach good representativeness. Similar reasons could also apply to other EU/EEA countries that do not have a monitoring system in place yet.

On the other hand, the 12 countries with one or more national monitoring systems represent an opportunity to launch EARS-Vet.

### Description and Analysis of National Monitoring Systems for Antimicrobial Resistance in Bacterial Pathogens of Animals

Our survey showed the diversity of organizations and operations of monitoring systems and may be particularly valuable to countries planning to establish or improve their system, by learning from the experience of other countries. As all systems had their own strengths and weaknesses, a lesson to learn may be that there is no single best way to monitor AMR in bacterial pathogens of animals and that each system needs to be adapted to its national context, capacities and objectives.

Despite their differences, many systems shared common weaknesses, e.g., in data management and representativeness, and common threats, such as economic vulnerability and data access. Moreover, only two monitoring systems underwent evaluations, although this is an important practice to allow more transparent interpretation of outputs, more objective decision-making and resource allocation, as well as improvements in system design and enhanced acceptance of system outputs by stakeholders (Peyre et al., 2019). However, our analysis showed that solutions exist to these frequent challenges. For instance, several national monitoring systems succeeded in collaborating with the private sector to collect more AST data and improve representativeness. Thus, public-private partnerships appear as an opportunity to improve representativeness and tackle the threat of data access, attributed to the development of more competitive private diagnostic laboratories, whose AST results are not currently captured by the monitoring system. In France, a “win-win approach” was developed where laboratories (including private ones) share their data in exchange of technical support and free PT. It enabled to build a very large network of public and private laboratories (Mader et al., 2021b). In Denmark, good collaboration between the industry (SEGES) and the public sector (DTU) illustrates that both may experience benefits with the animal industry showing transparency, without “hiding” potential AMR problems, and the public sector being able to publish and analyze data in national reports.

Our review also contributed to the consolidation of a preliminary EARS-Vet network, thanks to numerous interactions with key experts from national monitoring systems. Such a network represents an opportunity to address frequent challenges of monitoring systems collectively and in a coordinated way.

### Moving Toward the European Antimicrobial Resistance Surveillance Network in Veterinary Medicine

This review generated key information to advise the development of AMR monitoring in clinical animal isolates in Europe and more specifically to advance on the definition of the EARS-Vet framework. Indeed, EARS-Vet would need to collect accurate, representative and harmonized AST results produced and communicated by national monitoring systems.

First, we showed that coordinating institutions own their AST data in all but two countries. This should facilitate the development of an EARS-Vet data sharing agreement. Moreover, all systems collect quantitative AST results, namely MICs for broth microdilution and inhibition zone diameters for disk diffusion, which would facilitate the re-calculation of proportions of non-wild type (or resistant) isolates, should interpretation criteria change over time.

Second, most national monitoring systems are based on laboratories that are accredited on the AST technique and/or participate in PT and all of them implement quality assurance. Therefore, the capacity of national systems to produce accurate AST results is considered high. Still, the development of a European PT, as done in the framework of EARS-Net, should be considered.

Third, data representativeness is a frequent weakness of national monitoring systems, linked to the passive monitoring procedure they follow. However, representativeness is rarely assessed in practice. In the Netherlands, it was shown that passive monitoring led to biased estimates of resistance to clindamycin, chloramphenicol, erythromycin and kanamycin in *Staphylococcus pseudintermedius* (Broens et al., 2019). The same result was obtained in Denmark for clindamycin (Larsen et al., 2015). On the other hand, a social-science study in France based on 66 interviews of veterinary practitioners showed that culture and AST are carried out in nearly all suspicions of bacterial infections in chicken and turkey production, limiting the possibility for bias in these animal species (Bourély et al., 2018). More investigations on the value of passive monitoring approaches are needed and common indicators of representativeness should be developed to advise the comparability of AMR data between countries (Mader et al., 2021a). Finally, 9 from 15 monitoring systems do not collect information on reasons for requesting AST or if sampled animals have already been treated with an antimicrobial. Common efforts are needed by countries to collect this information more consistently to address important sources of bias.

Regarding harmonization, a major challenge is the diversity of AST procedures and interpretation criteria used by countries and the lack of many veterinary clinical breakpoints and ECOFFs. Therefore, it is necessary to define EARS-Vet standards and a harmonization strategy. EARS-Vet could follow the example of EARS-Net by accepting quantitative AST results produced by both disk diffusion and broth microdilution. However, for antimicrobials for which disk diffusion is not accurate (e.g., colistin), EARS-Vet should accept only MIC data. Regarding the interpretation of AST results, EU-JAMRAI experts suggested using EUCAST ECOFFs, although more countries currently use CLSI veterinary clinical breakpoints. The reason for this is that for many drug-bug combinations, animal- and infection-specific clinical breakpoints are missing and will likely not be established in the short term. Moreover, EUCAST ECOFFs enable early detection of changing AMR trends and would facilitate the integration of EARS-Vet with the EFSA monitoring in zoonotic and indicator bacteria from healthy food-producing animals. Although many EUCAST ECOFFs are currently missing for the combinations to be monitored in EARS-Vet (Mader et al., 2022), ECOFFs remain easier to produce than clinical breakpoints. As EUCAST and CLSI are based on the same broth microdilution technique [at least for non-fastidious organisms: ISO 20776-1 (2019), which represent the majority of the bacterial species included in the EARS-Vet scope (Mader et al., 2022)], most systems would not need to change their AST procedures to enable AST interpretation with EUCAST ECOFFs. However, it should still be explored if antimicrobial concentrations currently tested in national monitoring systems would allow interpretation with EUCAST ECOFFs, which may not be the case when only limited dilution ranges are tested. Regarding laboratories which use disk diffusion, they would have to adapt their method to the EUCAST methodology so that harmonized AST data can be collected by EARS-Vet. Although EUCAST ECOFFs would be the reference interpretation criteria for EARS-Vet, it would remain possible to calculate proportions of clinical resistance for the specific combinations of animal species/bacterial species/specimen/antimicrobial where a CLSI clinical breakpoint is available, for the subset of countries following the CLSI standard, with the aim to provide more relevant information in a clinical perspective.

Thus, the current landscape of AMR monitoring in the EEA showed many gaps but also an opportunity to launch EARS-Vet, as 15 national monitoring systems were already in place in 2020 with strong capacity in AST. Thanks to a thorough understanding of their practices, a pragmatic harmonization strategy could be proposed for AST. This work, combined with the definition of the EARS-Vet objectives (Mader et al., 2021a) and scope (Mader et al., 2022), provides key elements of the EARS-Vet framework, to support the generation of stronger evidence on AMR levels in bacterial pathogens of animals in Europe.

## Data Availability Statement

The original contributions presented in the study are included in the article/Supplementary Material, further inquiries can be directed to the corresponding author/s.

## Eu-Jamrai Partners

Evelyne Jouvin-Marche, Marie-Cécile Ploy, Sadika Bernard, Yohann Lacotte, Céline Pulcini, Marielle Bouqueau, Anton Hlava, Vera Buhmann, Eline Vandael, Lieven De Raedt, Blazenka Hunjak, Bojana Raickovic, Barbora Macková, Helena Žemlièková, Ute Wolff Sönksen, Sissel Skovgaard, Jüri Ruut, Ljudmila Linnik, Arina Zanuzdana, Nadiya Oezcelik, Flora Kontopidou, Mariana Tsana, Alkiviadis Vatopoulos, Achilleas Gikas, Aimilia Magkanaraki, Alessandra Cozza, Domenico Martinelli, Rosa Prato, Annalisa Pantosti, Francesca Prestinaci, Luca Busani, Roberta Creti, Uga Dumpis, Asta Dambrauskiene, Astra Vitkauskiene, Silvija Kiveryte, Agniete Mazzella, Rolanda Valinteliene, Robertas Petraitis, Jasper Claessen, Rosa Perán, Elma Smeets, Pita Spruijt, Svein Høegh Henrichsen, Christine Årdal, Mari Molvik, Oliver Kacelnik, Anne Margrete Urdahl, Dorota Żabicka, Sérgio Gomes, Razvan Ciortea, Maja Šubelj, Nina Jemec, António López Navas, Cristina Muñoz Madero, Laura Alonso Irujo, María Santacreu García, Gloria Oliva, Marta Massanés, Elena Ferragut, Eusebi Castaño, Casimiro Jiménez Guillén, Marisol Fragoso, Germán Peñalva, José Miguel Cisneros, Milena Estevez, Sophie Monteau, María José González de Suso, Pilar Gallego Berciano, Daniele Alioto, Lotta Edman, Axana Haggar, Elisabet Lindal, Jakob Ottoson, Anna Nordenfelt, Björn Bengtsson, Patriq Fagerstedt, Birgitta Lytsy, Jean Yves Madec, Lucie Collineau, Rodolphe Mader, Anne Berger-Carbonne, Karl Pedersen, Monika Larsson, Annicka Reuss, Anne Swalue, Cristina Portugal, Hans Fredrik Wilhelmsen, Jesús Oteo-Iglesias, Janicke Fischer, Helga Katharina Haug, Maria Da Graca Freitas, and María del Pilar López Acuña.

## Author Contributions

RM coordinated the overall study, led the questionnaire and SWOT analyses in the 11 countries, coordinated the group discussions to develop the initial EARS-Vet framework, and drafted the initial manuscript. LC analyzed the SWOT results and drafted the corresponding section. J-YM, CMM, and J-PA supervised the overall work. CMM, BA, CB, EMB, PC-R, PD, WF, AH, JH, HK, MN, KP, TP, LP, RS, CTJ, AMU, J-YM, and J-PA contributed to the physical and virtual meetings to answer the questionnaires and SWOT analyses. All authors have contributed to developing the methodology, and joined discussions, which led to initial agreements on the EARS-Vet framework, reviewed the draft for important intellectual content and approved the final version.

## Author Disclaimer

The views expressed in this publication are those of the authors and do not necessarily reflect the opinions of consulted experts and organizations.

## Conflict of Interest

The authors declare that the research was conducted in the absence of any commercial or financial relationships that could be construed as a potential conflict of interest.

## Publisher’s Note

All claims expressed in this article are solely those of the authors and do not necessarily represent those of their affiliated organizations, or those of the publisher, the editors and the reviewers. Any product that may be evaluated in this article, or claim that may be made by its manufacturer, is not guaranteed or endorsed by the publisher.

## Footnotes

1
https://eu-jamrai.eu/

## References

[B1] ARSIA (2017). Antibiogrammes: Rapport d’activités et résultats de l’ARSIA. Ciney: Arsia.

[B2] Bourély C. Fortané N. Calavas D. Leblond A. Gay É (2018). Why do veterinarians ask for antimicrobial susceptibility testing? A qualitative study exploring determinants and evaluating the impact of antibiotic reduction policy. *Prev. Vet. Med.* 159 123–134. 10.1016/j.prevetmed.2018.09.009 30314775

[B3] Broens E. M. Gonggrijp M. Biesheuvel M. van Hout J. (2019). *Comparison of Antimicrobial Susceptibility in Staphylococci from First-Time Canine Pyoderma Cases Versus Cases with an Unknown Treatment History. in (Utrecht), 1.* Available online at: https://www.uu.nl/sites/default/files/poster_icohar.pdf (accessed March 9, 2022).

[B4] Bundesministerium der Justiz und für Verbraucherschutz (1976). *Medicinal Products Act in the Version Published on December 12, 2005 (BGBl. I p. 3394), as Last Amended by Article 5 of the Act of December 9, 2020 (BGBl. I p. 2870).* Available online at: https://www.gesetze-im-internet.de/amg_1976/BJNR024480976.html (accessed March 24, 2022).

[B5] Calba C. Goutard F. L. Hoinville L. Hendrikx P. Lindberg A. Saegerman C. (2015). Surveillance systems evaluation: a systematic review of the existing approaches. *BMC Public Health* 15:448. 10.1186/s12889-015-1791-5 25928645 PMC4418053

[B6] Dierengezondheidszorg Vlaanderen (2019). *Antibioticaresistentie - Evolutie tot eind.* Available online at: https://www.dgz.be/publicaties/antibioticaresistentie-evolutie-tot-eind-2019 (accessed March 14, 2021)

[B7] European Centre for Disease Prevention and Control (2016). *EU Protocol for Harmonised Monitoring of Antimicrobial Resistance in Human Salmonella and Campylobacter Isolates.* Available online at: https://www.ecdc.europa.eu/sites/portal/files/media/en/publications/Publications/antimicrobial-resistance-Salmonella-Campylobacter-harmonised-monitoring.pdf (accessed October 25, 2020)

[B8] European Centre for Disease Prevention and Control (2020). *Antimicrobial Resistance in the EU/EEA (EARS-Net) - Annual Epidemiological Report 2019.* Available online at: https://www.ecdc.europa.eu/sites/default/files/documents/surveillance-antimicrobial-resistance-Europe-2019.pdf (accessed January 7, 2021)

[B9] European Centre for Disease Prevention and Control and Directorate-General for Health and Food Safety (2019). *Final Joint Report In Respect Of A One Health Country Visit To Ireland From 07 October 2019 To 11 October 2019 To Discuss Policies Relating To Antimicrobial Resistance.* Available online at: https://www.ecdc.europa.eu/sites/default/files/documents/antimicrobial-resistance-one-health-ireland-country-visit.pdf (accessed March 22, 2021)

[B10] European Commission (2017). *A European One Health Action Plan against Antimicrobial Resistance.* Brussels: European Commission.

[B11] European Food Safety Authority and European Centre for Disease Prevention and Control (2021). *The European Union Summary Report on Antimicrobial Resistance in Zoonotic and Indicator Bacteria from Humans, Animals and Food in 2018/2019.* Available online at: https://efsa.onlinelibrary.wiley.com/doi/epdf/10.2903/j.efsa.2021.6490 (accessed March 9, 2022). 10.2903/j.efsa.2018.5182 PMC7009656 32625816

[B12] Ferreira J. P. Stärk K. (2017). Antimicrobial resistance and antimicrobial use animal monitoring policies in Europe: where are we? *J. Public Health Pol.* 38 185–202. 10.1057/s41271-017-0067-y 28533531

[B13] Government of the Czech Republic (2019). Government of the Czech Republic Resolution no.‘75 on the Action Plan of the National Antibiotic Programme of the Czech Republic for Period 2019–2022. Prague: Government of the Czech Republic.

[B14] Larsen R. Boysen L. Berg J. Guardabassi L. Damborg P. (2015). Lincosamide resistance is less frequent in Denmark in *Staphylococcus pseudintermedius* from first-time canine superficial pyoderma compared with skin isolates from clinical samples with unknown clinical background. *Vet. Dermatol.* 26 202–205, e43–e44. 10.1111/vde.12209 25891140

[B15] Mader R. Bourély C. Amat J.-P. Broens E. M. Busani L. (2022). Defining the scope of the European antimicrobial resistance surveillance network in veterinary medicine (EARS-Vet): a bottom-up and one health approach. *J. Antimicrob. Chemother.* 77 816–826. 10.1093/jac/dkab462 35022739 PMC8864999

[B16] Mader R. Damborg P. Amat J.-P. Bengtsson B. Bourély C. Broens E. M. (2021a). Building the European antimicrobial resistance surveillance network in veterinary medicine (EARS-Vet). *Euro Surveill.* 26:2001359. 10.2807/1560-7917.ES.2021.26.4.2001359 33509339 PMC7848785

[B17] Mader R. Jarrige N. Haenni M. Bourély C. Madec J.-Y. Amat J.-P. (2021b). OASIS evaluation of the French surveillance network for antimicrobial resistance in diseased animals (RESAPATH): success factors underpinning a well-performing voluntary system. *Epidemiol. Infect.* 149:e104. 10.1017/S0950268821000856 33877045 PMC8161364

[B18] Mader R. Muñoz Madero C. Aasmäe B. Bourély C. Broens E. M. Busani L. (2021c). *Review and Analysis of National Monitoring Systems for Antimicrobial Resistance in Animal Bacterial Pathogens in Europe: a Basis for the Development of the European Antimicrobial Resistance Surveillance Network in Veterinary Medicine (EARS-Vet).* Zenodo. 10.5281/zenodo.5205371 PMC9023068 35464909

[B19] Mesa Varona O. Chaintarli K. Muller-Pebody B. Anjum M. F. Eckmanns T. Norström M. (2020). Monitoring antimicrobial resistance and drug usage in the human and livestock sector and foodborne antimicrobial resistance in six european countries. *IDR Volume* 13 957–993. 10.2147/IDR.S237038 32308439 PMC7140725

[B20] Peyre M. Hoinville L. Njoroge J. Cameron A. Traon D. Goutard F. (2019). The RISKSUR EVA tool (Survtool): a tool for the integrated evaluation of animal health surveillance systems. *Prev. Vet. Med.* 173:104777. 10.1016/j.prevetmed.2019.104777 31731037

[B21] Renault V. (2021). “Chapter 3. assessing community needs and resources,” in *Section 14. SWOT Analysis: Strengths, Weaknesses, Opportunities, and Threats | Main Section* (Community Tool Box). Available online at: https://ctb.ku.edu/en/table-of-contents/assessment/assessing-community-needs-and-resources/swot-analysis/main (accessed March 13, 2021)

[B22] Schrijver R. Stijntjes M. Rodríguez-Baño J. Tacconelli E. Babu Rajendran N. Voss A. (2017). Review of antimicrobial resistance surveillance programmes in livestock and meat in EU with focus on humans. *Clin. Microbiol. Infect.* 24 577–590. 10.1016/j.cmi.2017.09.013 28970159

