# Supplementary material for: Review and Analysis of National Monitoring Systems for Antimicrobial Resistance in Animal Bacterial Pathogens in Europe: A Basis for the Development of the European Antimicrobial Resistance Surveillance Network in Veterinary Medicine (EARS-Vet)
Source: Front Microbiol. 2022 Apr 7;13:838490. doi: 10.3389/fmicb.2022.838490 (PMC9023068; doi:10.3389/fmicb.2022.838490)
Supplement: Supplementary file 1 [file Data_Sheet_1.zip › Table S2.docx]

Supplementary Table S2: Name, regulation, organization and funding sources of 15 national monitoring systems for antimicrobial resistance in bacterial pathogens of animals (2020 as the reference year)

| **Country** | **Name of monitoring system** | **Regulated system** | **Funding sources** | **Steering committee** | **Composition of the steering committee (if present)** | **Coordinating institutions** | **Status of coordinating institutions** |
| --- | --- | --- | --- | --- | --- | --- | --- |
| **Finland** | FINRES-Vet | No | Regular budget of coordinating institutions | Absent | NA | Finnish Food Authority and University of Helsinki | Public |
| **Sweden** | Svarm | No | Regular budget of the coordinating institution | Absent | NA | National Veterinary Institute | Public |
| **Sweden** | SvarmPat | No | Dedicated budget enveloped for specific projects | Absent | NA | National Veterinary Institute and Farm & Animal Health (private farming company); additional organizations may be involved depending on project | Public and private |
| **The Czech Republic** | CZ NMTP | Yes | Regular budget of coordinating institutions; AST data production supported by the Czech government, which subsidizes AST to veterinarians. | Present | State Veterinary Administration, State Veterinary Institutes, Veterinary Research Institute, Institute for State Control of Veterinary Biologicals and Medicines, Chamber of Veterinary Surgeons, farmers' associations and National Public Health Institute | State Veterinary Institute in Jihlava | Public-private institute |
| **Norway** | NORM-VET | No | Regular budget of coordinating institutions | Absent | NA | Norwegian Food Safety Authorities and the Norwegian Veterinary Institute | Public |
| **Denmark** | DTU/VFA* | No | Regular budget of the coordinating institution and supported by dedicated public funding. | Present | Danish Veterinary and Food Administration*^/^** | Technical University of Denmark and Danish Veterinary and Food Administration | Public |
| **Denmark** | UC* | No | Regular budget of the coordinating institution | Absent | NA | University of Copenhagen | Public |
| **Denmark** | SEGES* | No | Regular budget of the coordinating institution | Absent | NA | Agricultural knowledge and innovation center (SEGES) | Private |
| **The Netherlands** | UU* | No | Entirely supported by dedicated public funding. | Absent | NA | University of Utrecht | Public |
| **The Netherlands** | GD Animal Health Surveillance System | No | Hours for collecting, processing and reporting data are subsidized via the Dutch animal Health monitoring; public financial support to pathological examinations on farm animals, which are an important source of AST results; financial support from producer/interbranch organizations and government. | Present | Ministry of agriculture, representatives of different livestock sectors (producers, feed, veterinarians etc.) and Royal GD | Royal GD | Private |
| **Germany** | GE*RM*-Vet | Yes | Regular budget of the coordinating institution; Specific public funding allocated to the Federal Office of Consumer Protection and Food Safety to perform AST. | Absent | NA | Federal Office of Consumer Protection and Food Safety | Public |
| **Ireland** | DAFM* | No | Regular budget of the coordinating institution | Absent | NA | Department of Agriculture, Food and the Marine | Public |
| **Spain** | SEVAE | No | Regular budget of coordinating institutions; AST data production supported by the Spanish government, which subsidizes AST to veterinarians. | Present | Spanish Agency of Medicines and Medical Products, Universidad de Veterinaria de Lérida, Laboratorio de análisis GSP | Spanish Agency of Medicines and Medical Products | Public |
| **Estonia** | VFL/ULS* | No | Regular budget of the coordinating institution | Absent | NA | Veterinary and Food Laboratory, University of Life Sciences | Public |
| **France** | RESAPATH | No | Regular budget of the coordinating institution | Present | Public and private diagnostic laboratories, Ministry in charge of Agriculture, veterinary professional organizations, French Agency for Food, Environmental and Occupational Health & Safety | French Agency for Food, Environmental and Occupational Health & Safety | Public |

*Acronyms of coordinating institutions were used to identify monitoring systems without official name for the purpose of this study. **During 2020, under the administration of the VFA, the coordination of this monitoring system has been gradually taken over by the Statens Serum Institut and the University of Copenhagen. DTU does however still supply data, e.g. for cattle pathogens. AST: Antimicrobial Susceptibility Testing; NA: Not Applicable.
