# Supplementary material for: Review and Analysis of National Monitoring Systems for Antimicrobial Resistance in Animal Bacterial Pathogens in Europe: A Basis for the Development of the European Antimicrobial Resistance Surveillance Network in Veterinary Medicine (EARS-Vet)
Source: Front Microbiol. 2022 Apr 7;13:838490. doi: 10.3389/fmicb.2022.838490 (PMC9023068; doi:10.3389/fmicb.2022.838490)
Supplement: Supplementary file 1 [file Data_Sheet_1.zip › Table S4.docx]

Supplementary Table S4: Description of the laboratory networks of 15 national monitoring systems for antimicrobial resistance in bacterial pathogens of animals (2020 as the reference year)

| **Country** | **Name of monitoring system** | **Presence of a network of field diagnostic laboratories** | **Number of networked laboratories** | **Status of networked laboratories** | **Presence of a central laboratory (if there is a network)** | **Laboratory activities of the central laboratory** | **Is the central laboratory (or the unique laboratory in the absence of network) the National Reference Laboratory for AMR in the animal sector?** |
| --- | --- | --- | --- | --- | --- | --- | --- |
| **Finland** | FINRES-Vet | Yes | 5 | Public | Yes | Diagnostic activities, reference activities and complementary molecular analyses. | Yes |
| **Sweden** | Svarm | No | NA | NA | NA | NA | Yes |
| **Sweden** | SvarmPat | No | NA | NA | NA | NA | Yes |
| **The Czech Republic** | CZ NMTP | Yes | 3 | Public/Private | Yes | Diagnostic activities, reference activities, complementary molecular analyses and central strain collection of all isolates. | No |
| **Norway** | NORM-VET | Yes | 2 | Public or private | Yes | Diagnostic activities, reference activities, collecting isolates from one collaborating private field laboratory, isolates being re-identified and re-tested by AST and complementary molecular analyses. | Yes |
| **Denmark** | DTU/VFA* | Yes | 2 | Public or private | Yes | Confirmation of diagnostics (e.g. MALDI-TOF) and serotyping of some pathogens | No |
| **Denmark** | UC* | No | NA | NA | NA | NA | No |
| **Denmark** | SEGES* | No | NA | NA | NA | NA | No |
| **The Netherlands** | UU* | No | NA | NA | NA | NA | No |
| **The Netherlands** | GD Animal Health Surveillance System | No | NA | NA | NA | NA | No |
| **Germany** | GE*RM*-Vet | Yes | 30 | Public or private | Yes | Diagnostic activities (not sure?), reference activities, collection of isolates from field laboratories which are all re-identified and re-tested by AST and complementary molecular analyses. | No |
| **Ireland** | DAFM* | Yes | 6 | Public | Yes | Diagnostic activities, reference activities, collection of isolates from field laboratories when they exhibit specific phenotypes which are re-identified and re-tested by AST and complementary molecular analyses. | Yes |
| **Spain** | SEVAE | Yes | 22 | Public or private | No | NA | NA |
| **Estonia** | VFL/ULS* | No | NA | NA | NA | NA | Yes |
| **France** | RESAPATH | Yes | 71 | Public or private | Yes (two central laboratories) | Reference activities, collection of isolates from field laboratories when they exhibit specific phenotypes which are the re-identified and re-tested by AST, complementary molecular analyses and laboratory trainings. | No |

*Acronyms of coordinating institutions were used to identify monitoring systems without official name for the purpose of this study (see Supplementary Table S2).

AST: Antimicrobial Susceptibility Testing; NA: Not Applicable; MALDI-TOF: Matrix Assisted Laser Desorption Ionization - Time of Flight
