# Supplementary material for: Review and Analysis of National Monitoring Systems for Antimicrobial Resistance in Animal Bacterial Pathogens in Europe: A Basis for the Development of the European Antimicrobial Resistance Surveillance Network in Veterinary Medicine (EARS-Vet)
Source: Front Microbiol. 2022 Apr 7;13:838490. doi: 10.3389/fmicb.2022.838490 (PMC9023068; doi:10.3389/fmicb.2022.838490)
Supplement: Supplementary file 1 [file Data_Sheet_1.zip › Table S5.docx]

Supplementary Table S5: Laboratory techniques and standards used in 15 national monitoring systems for antimicrobial resistance in bacterial pathogens of animals (2020 as the reference year)

| **Country** | **Name of surveil-lance program** | **Same bacterial species identification method for all labs** | **Method for bacterial identification** | **All labs follow the same AST method** | **AST method** | **AST standard** | **Primary interpretation criteria (when available)** | **AST accreditation** | **Labs participate in PT on AST** | **Phenotypes routinely confirmed at molecular level** |
| --- | --- | --- | --- | --- | --- | --- | --- | --- | --- | --- |
| **Finland** | FINRES-Vet | Yes | MALDI-TOF | No | BMD for farm animals and disk diffusion for companion animals | CLSI | CBP | Yes, for FFA labs | Yes, all labs participate in VETQAS® PT | MRSA, MRSP, colistin resistance and carbapenem resistance by PCR |
| **Sweden** | Svarm | Yes (only one lab) | MALDI-TOF | Yes (only one lab) | BMD | EUCAST | ECOFF | Yes | Yes | MRSA, MRSP, colistin resistance, carbapenem resistance, ESBL and AmpC confirmed by PCR and/or WGS |
| **Sweden** | SvarmPat | Yes (only one lab) | MALDI-TOF | Yes (only one lab) | BMD | EUCAST | ECOFF | Yes | Yes | MRSA, MRSP, colistin resistance, carbapenem resistance, ESBL and AmpC confirmed by PCR and/or WGS |
| **The Czech Republic** | CZ NMTP | Yes (checked by PT) | MALDI-TOF | Yes | BMD | CLSI | CBP | Yes | Yes | MRSA and ESBL confirmations by PCR |
| **Norway** | NORM-Vet | Yes (the central lab re-identifies all bacterial species) | MALDI-TOF | Yes (the central lab re-tests all bacterial species) | BMD | EUCAST | ECOFF | Yes | No (All isolates are re-tested centrally) | MRSA, MRSP, colistin resistance, vancomycin resistance, carbapenem resistance, ESBL and AmpC confirmed by PCR and/or WGS |
| **Denmark** | DTU/VFA* | Yes | MALDI-TOF | Yes | BMD | CLSI | CBP | Yes | Yes | Project dependent |
| **Denmark** | UC* | Yes (only one lab) | MALDI-TOF | Yes (only one lab) | BMD | CLSI | CBP | No | No | None |
| **Denmark** | SEGES* | Yes (only one lab) | Colony morphology, selective agars, or agglutinations. If necessary, MALDI-TOF is carried out at DTU. | Yes (only one lab) | BMD | CLSI | CBP | Yes | Yes | None |
| **The Netherlands** | UU* | Yes (only one lab) | MALDI-TOF | Yes (only one lab) | BMD | CLSI | CBP | No | Yes (PT organized by the NRL) | MRSA and MRSP confirmations by PCR |
| **The Netherlands** | GD Animal Health Surveillance System | Yes (only one lab) | MALDI-TOF | Yes (only one lab) | BMD | CLSI | CBP | No | Yes (PT organized by the NRL and VetQAS® PT) | MRSA confirmation by PCR |
| **Germany** | GE*RM*-Vet | Yes (the central lab re-identifies all bacterial species) | MALDI-TOF | Yes (the central lab re-tests all isolates) | BMD | CLSI | CBP | Yes | No (All isolates are re-tested centrally) | MRSA, colistin resistance and ESBL confirmed by PCR |
| **Ireland** | DAFM* | Yes (ensured by an SOP and checked by PT) | API galleries | Yes | Disk diffusion (BMD to confirm specific phenotypes) | CLSI | CBP | No | Yes (VETQAS® PT) | MRSA, colistin resistance and ESBL confirmed by WGS |
| **Spain** | SEVAE | Yes (ensured by a signed agreement) | MALDI-TOF | Yes | BMD | CLSI | CBP | No | No | None |
| **Estonia** | VFL/ULS* | Yes (only one lab) | API galleries or MALDI-TOF | Yes (only one lab) | Disk diffusion (BMD to test colistin resistance) | CLSI | CBP | Yes | No | MRSA and colistin resistance confirmed by PCR |
| **France** | RESAPATH | No | API galleries or MALDI-TOF | Yes | Disk diffusion | CA-SFM | ECOFF | Only some labs | Yes (PT organized by ANSES) | MRSA, MRSP, colistin resistance, carbapenem resistance and ESBL confirmed by PCR and/or WGS |

*Acronyms of coordinating institutions were used to identify monitoring systems without official name for the purpose of this study (see Supplementary Table S2).

AMR: Antimicrobial Resistance; BMD: Broth Microdilution; ANSES: French Agency for Food, Environmental and Occupational Health and Safety; API: Analytical Profile Index; AST: Antimicrobial Susceptibility Testing; CA-SFM: Antibiogram Committee of the French Society of Microbiology; CBP: Clinical Breakpoints; CLSI: Clinical & Laboratory Standards Institute; ECOFF: Epidemiological Cut-Off Values; ESBL: Extended Spectrum Beta-Lactamase; EUCAST: European Committee on Antimicrobial Susceptibility Testing; FFA: Finish Food Authority; Lab: Laboratory; MALDI-TOF: Matrix Assisted Laser Desorption Ionization - Time of Flight; MRSA: Methicillin-Resistant *Staphylococcus aureus*; MRSP: Methicillin-Resistant *Staphylococcus pseudintermedius*; NRL: National Reference Laboratory; PCR: Polymerase Chain Reaction; PT: Proficiency Testing; SOP: Standard Operating Procedure; UH: University of Helsinki; WGS: Whole Genome Sequencing.
