# Supplementary material for: Review and Analysis of National Monitoring Systems for Antimicrobial Resistance in Animal Bacterial Pathogens in Europe: A Basis for the Development of the European Antimicrobial Resistance Surveillance Network in Veterinary Medicine (EARS-Vet)
Source: Front Microbiol. 2022 Apr 7;13:838490. doi: 10.3389/fmicb.2022.838490 (PMC9023068; doi:10.3389/fmicb.2022.838490)
Supplement: Supplementary file 1 [file Data_Sheet_1.zip › Table S6.docx]

Supplementary Table S6: Collected data in 15 national monitoring systems for antimicrobial resistance in bacterial pathogens of animals

| **Country** | **Name of surveillance program** | **Surveillance data collected, apart from bacterial species, antimicrobial and AST result** | **Volume of isolates included in the monitoring (exact number and year or approximate range)** |
| --- | --- | --- | --- |
| **Finland** | FINRES-Vet | Animal species, breed, age, sex, date of sampling, specimen, if the animal was having an antimicrobial treatment at time of sampling and if yes, what antimicrobial. | 3650-3950 |
| **Sweden** | Svarm | Animal species, date, specimen and production type (dairy/beef) for cattle and poultry (broiler/layer, submitting veterinarian, owner/herd registration number if applicable. | 4000-6000 |
| **Sweden** | SvarmPat | Project-dependent. | Project-dependent |
| **Czech Republic** | CZ NMTP | AST date, laboratory identification, sample regional identification, epidemiological unit, animal species, production type and specimen. | 1300-1600  (for the period 2017-2020) |
| **Norway** | NORM-VET | Animal species, gender, age, production type, epidemiological unit, veterinarian or veterinary clinic, owner and owner location, specimen, disease or reason for asking an AST, date, identification number | 200-600 |
| **Denmark** | DTU/VFA* | Date, farm identification number, herd number, animal species, specimen, date of sampling. | Project-dependent |
| **Denmark** | UC* | Animal species, specimen, disease or reason for asking an AST, date of sampling. | 600 |
| **Denmark** | SEGES* | Date, farm identification number, animal species, specimen, bacterial serotype (when relevant), date of sampling. | 500 |
| **The Netherlands** | UU* | Date, animal species, age, location, specimen, disease, previous treatment, travel history | 7800 (in 2020) |
| **The Netherlands** | GD Animal Health Surveillance System | Date, animal species, age, location, specimen, disease, production type, if the sample has been directly submitted or if it comes from a necropsy performed at Royal GD. For poultry: treatment history. | 8676 (2020) |
| **Germany** | GE*RM*-Vet | Animal species, specimen, age, production type, clinical signs, if the animal was having an antimicrobial treatment at time of sampling and if yes, what antimicrobial, farm postal code, sample date. | 2500-3000 |
| **Ireland** | DAFM* | Animal species, breed, age, sex, data, date of sampling, specimen, if the animal was having an antimicrobial treatment at time of sampling, production type (e.g. dairy or beef) | 7700 (2018) |
| **Spain** | SEVAE | Date, laboratory identifier, farm identifier, veterinary clinic identifier, regional identifier, epidemiological unit and animal species. | Not yet known at the time of writing as the monitoring system started in 2020. |
| **Estonia** | VFL/ULS* | Farm identifier, animal species, age, if the animal was having an antimicrobial treatment at time of sampling and if yes, what antimicrobial. | Project dependent |
| **France** | RESAPATH | Laboratory identifier, zip code and town of the farmer or companion animal owner, AST date, animal species, production type, age category, specimen and disease. | 55401 (in 2018) |

*Acronyms of coordinating institutions were used to identify monitoring systems without official name for the purpose of this study (see Supplementary Table S2).

AST: Antimicrobial Susceptibility Testing
