# Supplementary material for: Review and Analysis of National Monitoring Systems for Antimicrobial Resistance in Animal Bacterial Pathogens in Europe: A Basis for the Development of the European Antimicrobial Resistance Surveillance Network in Veterinary Medicine (EARS-Vet)
Source: Front Microbiol. 2022 Apr 7;13:838490. doi: 10.3389/fmicb.2022.838490 (PMC9023068; doi:10.3389/fmicb.2022.838490)
Supplement: Supplementary file 1 [file Data_Sheet_1.zip › Table S7.docx]

Supplementary Table S7: Communication activities of 15 national monitoring systems for antimicrobial resistance in bacterial pathogens of animals

| **Country** | **Name of the surveillance program** | **Communication tools** | **Frequency of surveillance report publication** | **Lag** **between AST data production and report publication** | **Target audience of communication activities** |
| --- | --- | --- | --- | --- | --- |
| **Finland** | FINRES-Vet | Surveillance reports, magazines for veterinarians and farmers | Once a year (since 2018) | 11 months (since 2018) | Finnish Food Authority experts, National Institute for Health and Welfare experts, Animal Health ETT, veterinary practitioners, farmers and journalists |
| **Sweden** | Svarm | Surveillance reports, factsheets, presentations at annual stakeholder meetings before the release of the report, presentations of results during lectures | Once a year | 6 months | National Veterinary Institute, professional associations producing treatment recommendations, individual veterinarians |
| **Sweden** | SvarmPat | Surveillance reports, factsheets, presentations at annual stakeholder meetings before the release of the report, presentations of results during lectures | Once a year (although some projects collect data for several years before reporting) | 6 months | National Veterinary Institute, professional associations producing treatment recommendations, individual veterinarians |
| **The Czech Republic** | CZ NMTP | Surveillance reports, magazines for veterinarians and farmers, presentations of results during lectures | Once a year | 6 months | Veterinary practitioners, farmers, national authorities, Czech pharmaceutical industry, researchers, One Health network |
| **Norway** | NORM-VET | Annual surveillance reports, annual meetings when delivering the report, meetings with the Norwegian Food Safety Authority and the industry, on NVI website (and sometimes in magazines for veterinarians or farmers) | Once a year | 9 months (however, isolates included in the report may have been collected over several years) | Norwegian Food Safety Authority, farming industry, Ministry in charge of Agriculture, researchers, veterinary practitioners |
| **Denmark** | DTU/VFA* | Surveillance reports and other publications | Once a year | 1 year | Veterinary practitioners, national authorities and stakeholders producing treatment policies/guidelines |
| **Denmark** | UC* | Surveillance reports and other publications | Every two years | 1-2 years | Veterinary practitioners, national authorities and stakeholders producing treatment policies/guidelines |
| **Denmark** | SEGES* | Surveillance reports and on their website | 4 times a year | 1 month for quarter reports on their website and 9 months for the joint report with DTU Vet | Veterinary practitioners, farmers, national authorities and stakeholders producing treatment policies/guidelines |
| **The Netherlands** | UU* | Surveillance reports to Ministries, presentations at meetings and conferences, in magazines for veterinarians, scientific papers | Once a year | 4-5 months | Veterinary practitioners, national authorities in charge of producing treatment guidelines and the government |
| **The Netherlands** | GD Animal Health Surveillance System | Surveillance reports, on their website, in scientific publications, in magazines for veterinarians or farmers | Every 3 or 6 months depending on the animal species | 4-5 months | Veterinary practitioners, government, pharmaceutical industry, national authorities in charge of producing treatment guidelines. |
| **Germany** | GE*RM*-Vet | Surveillance reports, at meetings and conferences, on their website, in scientific papers, in magazines for veterinarians and farmers | Once a year | 1 year | Veterinary practitioners, government, medicines agencies |
| **Ireland** | DAFM* | Surveillance reports and local meetings with veterinarians | Once a year | Usually <1 year | Veterinary practitioners, farmers and government |
| **Spain** | SEVAE | Planned to be in surveillance reports, on their website and on a mobile application | Planned to be once a year | Unknown yet | Private vets, farmers and national authorities |
| **Estonia** | VFL/ULS* | Surveillance reports sent to the Ministry of Rural Affairs. | Once a year | 5-6 months | National authorities (Veterinary and Food Board and State Agency of Medicines) |
| **France** | RESAPATH | Surveillance reports, annual RESAPATH meetings for the release of the report, scientific publications, on the RESAPATH website | Once a year | 11 months | French Agency for Food, Environmental and Occupational Health & Safety, Ministries in charge of Agriculture and Health, veterinarians, epidemiologists, microbiologists |

*Acronyms of coordinating institutions were used to identify monitoring systems without official name for the purpose of this study (see Supplementary Table S2).
