# Supplementary material for: Review and Analysis of National Monitoring Systems for Antimicrobial Resistance in Animal Bacterial Pathogens in Europe: A Basis for the Development of the European Antimicrobial Resistance Surveillance Network in Veterinary Medicine (EARS-Vet)
Source: Front Microbiol. 2022 Apr 7;13:838490. doi: 10.3389/fmicb.2022.838490 (PMC9023068; doi:10.3389/fmicb.2022.838490)
Supplement: Supplementary file 1 [file Data_Sheet_1.zip › Table S8.docx]

Supplementary Table S8: Performance indicators of the French surveillance network for antimicrobial resistance in diseased animals (RESAPATH) calculated every year (reference year 2020)

| Performance indicator | Target outcome |
| --- | --- |
| Number of AST results collected | Increasing or equal number compared to the previous year |
| Number of member laboratories | Increasing or equal number compared to the previous year |
| Proportion of member laboratories submitting their AST data to ANSES | At least 90% |
| Proportion of isolates received by ANSES, among all the isolates requested to member laboratories by ANSES | At least 50% |
| Proportion of isolates that have been received within 31 days from the request date, among received isolates | At least 80% |
| Proportion of AST results integrated in the RESAPATH database within four months after the AST date | At least 60% |
| A RESAPATH report has been produced | Yes |
| A one-day RESAPATH meeting has been organized | Yes |
| Proportion of laboratories participating in the one-day RESAPATH meeting | At least 65% |
| Updating frequency of the RESAPATH website | Average period between two updates below three months |
| Proportion of questions sent by laboratories to ANSES that are answered within 15 days | At least 60% |
| The steering committee has met | Yes |
| Proportion of laboratories participating in the proficiency testing organized by ANSES | At least 90% |
| Proportion of laboratories obtaining a score above or equal to 31/36 at the proficiency testing organized by ANSES | At least 95% |

ANSES : French Agency for Food, Environmental and Occupational Health & Safety ; AST : Antimicrobial Susceptibility Testing
